# Pure red cell aplasia secondary to rheumatoid arthritis: a case report

**DOI:** 10.1186/s13256-021-03141-5

**Published:** 2021-12-06

**Authors:** Suneth Weerasinghe, Parackrama Karunathilake, Udaya Ralapanawa, Thilak Jayalath, Shamali Abeygunawardena, Manel Rathnayaka

**Affiliations:** 1 Teaching Hospital, Peradeniya, Sri Lanka; 2 Department of Medicine, Faculty of Medicine, University of Peradeniya, Kandy, Sri Lanka; 3 Department of Pathology, Faculty of Medicine, University of Peradeniya, Kandy, Sri Lanka

**Keywords:** Hematologic manifestations—rheumatoid arthritis, Pure red cell aplasia, Rheumatoid arthritis, Cyclosporine A

## Abstract

**Background:**

Rheumatoid arthritis is a common autoimmune disease with many extra-articular manifestations. Pure red cell aplasia is a rare manifestation of rheumatoid arthritis and is sparsely documented in the literature, with a variable clinical outcome following immunosuppressive therapy.

**Case presentation:**

A 63-year-old Sinhalese female presented with transfusion-dependent anemia associated with deforming inflammatory arthritis. She also had leukopenia, right subclavian venous thrombosis, and generalized lymphadenopathy. The diagnosis of rheumatoid arthritis following initial clinical workup and additional blood and bone marrow investigations revealed pure red cell aplasia as a secondary manifestation of rheumatoid arthritis after excluding other secondary causes, such as infections, thymoma, thrombophilic conditions, and hematological malignancy. She responded well to oral prednisolone, cyclosporine A, and hydroxychloroquine, and she attained complete recovery in 2 months.

**Conclusion:**

Pure red cell aplasia is a disabling illness that may lead to transfusion-dependent anemia, which may occur due to rare extrapulmonary manifestation of rheumatoid arthritis. The diagnosis of pure red cell aplasia secondary to rheumatoid arthritis may be challenging where hematological investigations, including bone marrow biopsy, will aid in the diagnosis, and early diagnosis and treatment will bring about a better outcome.

## Background

Rheumatoid arthritis (RA) is a common immune-mediated, chronic, symmetrical, inflammatory disease that initially affects small joints, progressing to larger joints and eventually causing extra-articular manifestations involving the skin, eyes, heart, kidneys, and lungs [1, 2]. Pure red cell aplasia (PRCA) is a rare manifestation of RA, a hemopoietic disorder first reported in 1922, presented with normocytic normochromic anemia with severe reticulocytopenia and marked reduction or absence of erythroid precursors from the bone marrow [3, 4]. The clinical outcome of PRCA varies from complete remission to fatal, and depends upon the cause and the types of treatment [5]. Therefore, it imposes a diagnostic and therapeutic challenge when PRCA presents with immune-mediated peripheral cytopenias secondary to autoimmune diseases. We present a rare case of PRCA secondary to RA presented with bicytopenia where the patient attained complete recovery with immunosuppressive therapy.

## Case presentation

A 63-year-old Sinhalese female was admitted to the Teaching Hospital Peradeniya in March 2017 with progressive generalized malaise, fatigue, and shortness of breath on exertion for 1 week. She did not complain of orthopnea, paroxysmal nocturnal dyspnea, or any previous syncopal attacks. There was no history of tingling sensations of the extremities, recurrent infections, previous miscarriages, or any history suggestive of thrombotic episodes.

She also had a 7-year history of pain and swelling in multiple joints, starting from the right ankle joint and progressing into multiple large joints in a symmetrical pattern associated with morning stiffness, lasting for about 4 hours a day. Simultaneously, she had developed involvement of the small proximal joints of the bilateral hands for 5 years. She had taken some over-the-counter medications, including non-steroidal antiinflammatory drugs (NSAIDs), some local applications, and some native treatments to relieve these symptoms, which she had been using for several years. However, she did not have any history of taking disease-modifying antirheumatoid drugs (DMARDs) before this admission. She had taken some indigenous medicines from time to time for a few years; however, she was not aware of their ingredients. She was working as a housemaid abroad when she developed joint pains and noticed some deformities appearing in her hands towards the end of 2016, when she found it challenging to engage in day-to-day activities.

She did not have any previous history of bleeding manifestations, including melena. She attained menopause at the age of 51, and there was no history of postmenopausal bleeding. She consumed an average Sri Lankan rice-based diet with adequate calories and nutrients with three meals per day.

**Fig. 1:**
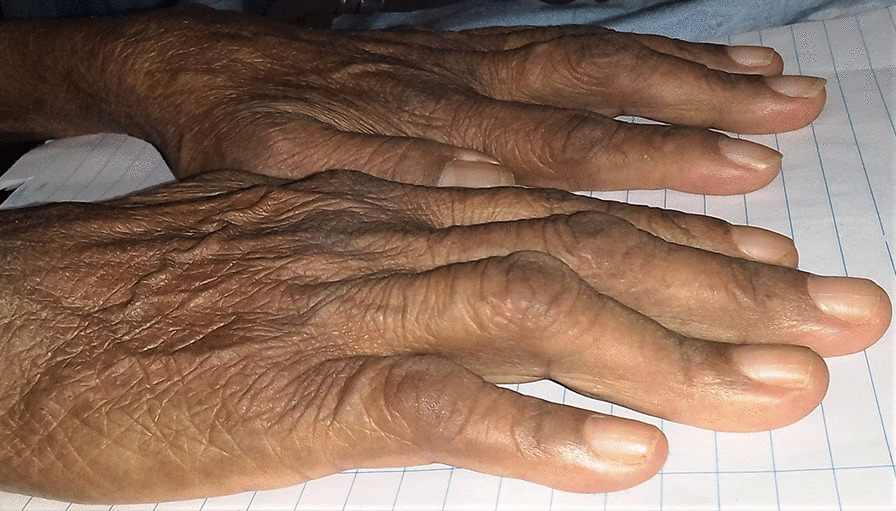
Boutonniere deformities in the left middle, right middle, and right ring fingers

At the current admission, she was severely pale, and there were boutonniere deformities in the left middle, right middle, and right ring fingers; however, there was no evidence of active joint inflammation in the form of swelling or erythema in the small joints of the hands (Fig. 1). There were no skin rashes, photosensitivity, tight skin on the fingers or face, cutaneous bleeding manifestations, oral ulcers, or alopecia, nor did she have any dependent edema. Her vital parameters were stable with a pulse rate of 84 beats per minute, blood pressure of 120/80 mmHg, and normal jugular venous pressure without any abnormal heart sounds. Other system examinations were unremarkable.

Initial investigations revealed a hemoglobin (Hb) level of 1.8 g/dL (12.1–15.1) with normal mean corpuscular volume (MCV) and corpuscular hemoglobin concentration (MCHC). White cell count was 2100/mm^3^ (4500–11000) (neutrophils 55% and lymphocytes 35%) with a platelet count of 169000/mm^3^ (150,000–450,000) and a reticulocyte count of 0.09% (0.5–2.5%) with negative direct and indirect Coombs tests. Her random blood sugar level was 5.7 mmol/L. Other investigations revealed erythrocyte sedimentation rate (ESR) of 55 mm/hour (0–20), C-reactive protein (CRP) 31.68 mg/L (< 3.0), aspartate aminotransferase (AST) 115 IU/L (9–32), alanine aminotransferase (ALT) 98 IU/L (19–25), total bilirubin 16.4 µmol/L (1.71–20.5), alkaline phosphatase (ALP) 223.8 IU/L (44–147), gamma-glutamyl transferase (GGT) 79.1 IU/L (8–38), serum albumin 22.9 g/dL (3.5–5.2), and albumin/globulin 0.97 (1.1–2.5). Her serum creatinine level was 65 µmol/L (52.2–91.9). Her blood electrolyte levels were serum sodium 137 mmol/L (135–145), potassium 4 mmol/L (3.5–5.0), magnesium 1.07 mmol/L (0.85–1.10), and corrected calcium 2.4 mmol/L (2.2–2.6). The electrocardiographic findings, 2D echocardiogram, urinalysis, clotting profile, and chest x-ray were normal.

She was then further investigated for an etiology for anemia, where the blood picture showed bicytopenia (leukopenia and anemia) with features more in favor of megaloblastic anemia. Due to the anemia, she was transfused with 6 pints of red cell concentrate (RCC) until Hb > 10 g/dL. Her serum iron level was 268.5 µg/dL (33–199) and total iron-binding capacity (TIBC) was within the normal range. The bone marrow biopsy showed markedly reduced erythroid lineage with increased granulopoiesis and normal megakaryopoiesis, and there was no evidence of lymphoma, leukemia, secondary deposit, or granuloma. Based on these findings, the diagnosis of PRCA was made, and leukopenia was thought to be immune in origin. She improved with corticosteroids and supportive treatments with iron, vitamin B12, and folic acid inward and was discharged, and in the 2-week follow-up she had no new complaints. Unfortunately, she did not attend any further clinic follow-ups.

She got readmitted in September 2017 with right upper limb swelling and was found to have right subclavian vein thrombosis. The lower limb venous duplex was typical, and there was no evidence of pulmonary embolism. The anticoagulation was done initially with enoxaparin and warfarin and subsequently continued with warfarin. The thrombophilia screen was negative with a regular lupus anticoagulant test. A contrast-enhanced computed tomography (CECT) of the chest, abdomen, and pelvis revealed bilateral axillary, level IV cervical, intercostal, iliac, and inguinal lymphadenopathy. The patient underwent an axillary lymph node biopsy, which showed preserved architecture and appearance consistent with reactive lymph nodes. Upper and lower gastrointestinal endoscopies were also done to exclude any evidence of malignancy or any other pathology that can cause blood loss, and both investigations turned out to be as expected. The patient was referred for a hematology opinion, where a bone marrow biopsy was suggested to exclude any hematological malignancy. However, the bone marrow biopsy showed hypercellular bone marrow with normal granulopoiesis, megakaryopoiesis, and absent erythropoiesis, where the possibility of lymphoma was excluded. The patient was treated with corticosteroids and hematinics, and showed a satisfactory response to improve the cytopenias. The upper limb swelling was also resolved after successful treatment with the anticoagulants. Since the patient’s primary concern was the symptoms due to venous thrombosis and cytopenias, the assessment of her joint symptoms was not carried out. She was discharged from the hospital with a follow-up planed in 2 weeks; however, she defaulted on the follow-up.

The subsequent admission was in June 2018 where she was found to have a vasculitic-type rash in the lower limb. A skin biopsy showed perivascular inflammatory infiltration predominantly by neutrophils and lymphocytes, with focal areas of fibrinoid necrosis suggestive of leukocytoclastic vasculitis. The patient was under investigation for secondary causes of PRCA during this presentation, where the human immune deficiency virus (HIV) screening, viral hepatitis screening (Hepatitis B surface antigen and Hepatitis C antibody), and parvovirus b19 serology revealed negative results. However, she fulfilled the American College of Rheumatology (ACR) criteria for rheumatoid arthritis (2) with positive rheumatoid factor level (347 IU/mL; normal range 0–20 IU/mL) and negative antinuclear antibody (ANA) and anti-double stranded DNA (dsDNA) levels. The anti-cyclic citrullinated peptide antibody (anti-CCP) level was not done as it was not affordable. Overall, this patient had transfusion-dependent anemia for 16 months, which warranted transfusion of 36 units of RCC.

A multidisciplinary team meeting was held with the participation of a consultant hematologist, and the diagnosis of PRCA secondary to rheumatoid arthritis was made. After that, she was started with oral cyclosporine A 250 mg daily (5 mg/kg/day), hydroxychloroquine 200 mg twice daily, and oral prednisolone 40 mg daily, which was tailed off over 1 month. She was then followed-up at the medical and hematology clinic, where her hemoglobin levels improved and increased without any requirement for further blood transfusions. Her joint pains resolved, and her general wellbeing was markedly improved. The regular monitoring with full blood counts revealed normal white cell counts and hemoglobin levels in 2 months.

## Discussion

Rheumatoid arthritis (RA) is an immune-mediated, chronic, symmetrical, inflammatory disease that initially affects small joints, progressing to larger joints, and eventually the skin, eyes, heart, kidneys, and lungs. These patients often develop destruction in the bones and cartilage of joints, and eventually, the tendons and ligaments weaken [1, 2]. RA should be considered in any patient with joint stiffness, pain, or swelling that persists for more than a few weeks [6]. RA presents with various extra-articular manifestations evolving over a few weeks to months, along with joint symptoms characterized by widespread, persistent synovitis, and positivity of autoantibodies to the Fc portion of immunoglobulin G, rheumatoid factor (RF), and anti-cyclic citrullinated peptide antibodies (ACPA) [2]. According to the 2010 American College of Rheumatology/European League Against Rheumatism (ACR/EULAR) classification criteria for rheumatoid arthritis, the diagnosis of RA can be made in patients who have at least one joint with definite clinical synovitis (swelling) not better explained by another disease. The classification criteria for RA are based on a scoring system considering four entities: joint involvement, serology including RF and ACPA levels, acute-phase reactants including CRP and ESR, and the duration of symptoms [7]. Our patient fulfilled the 2010 ACR/EULAR criteria for RA. Presentation of the RA can be very heterogeneous in some patients associated with other autoimmune diseases. PRCA associated with RA is reported in the literature though it is not common [8, 9].

PRCA is a hemopoietic disorder first reported in 1922. The whole mark of the disease is normocytic normochromic anemia with severe reticulocytopenia and marked reduction or absence of erythroid precursors from the bone marrow [3]. It can be primary (congenital) or acquired secondary to viral infections, autoimmune diseases, drugs, and toxins [10]. Other secondary causes of acquired PRCA include lymphoproliferative disorders, pregnancy, hematologic malignancies, and nonhematologic neoplasms, of which the association with thymoma is the best known [10]. There are numerous cases of PRCA that have been reported as a secondary manifestation of RA [4, 5, 11–17]. Among them, four cases had eosinophilic fasciitis along with PRCA [11]. Some cases have been reported with PRCA associated with both RA and parvovirus B19 infection, and there were several cases of PRCA associated with pregnancy and systemic-onset juvenile idiopathic arthritis [12, 14, 15]. One case has been reported of PRCA secondary to D penicillamine treatment in RA [17].

Patients with PRCA present with symptoms of severe anemia in the absence of hemorrhagic phenomena [18]. Because there is pure underproduction anemia in PRCA, the gradual decline in hemoglobin concentration allows some degree of adaptation, and symptoms may be less than expected for the degree of anemia [10]. Of course, patients with secondary PRCA may manifest the symptomatology of the associated syndrome [10]. The findings of the hematological investigations will be normochromic normocytic anemia with low reticulocyte count (less than 1%) with normal platelet count, leukocyte count, and leukocyte differentials [10, 18]. In the setting of concurrent inflammation, there may be some modest reduction in the total white blood count or a mild abnormality (either slightly high or slightly low) in the platelet count. There may also be a mild relative lymphocytosis [10]. The bone marrow examination will reveal a complete absence of erythroblasts with normal granulopoiesis and megakaryopoiesis [4, 17, 18]. As with all diagnostic examinations of the bone marrow for cytopenias, the materials should be collected for cellular immunology, cytogenetics, and clonal analysis of T cell receptors. Abnormal cytogenetics in the setting of a characteristic marrow for PRCA indicates the myelodysplastic variant of PRCA. If increased lymphocytes or plasma cells are present, they should be polyclonal in acquired immune PRCA. If clonal lymphocytes are present, it suggests PRCA secondary to an associated lymphoproliferative disorder. T cell receptor gene rearrangement studies should be performed routinely [19]. In all patients with marrows diagnostic of PRCA, B19 parvovirus testing should be performed, and in adults with PRCA with evidence of parvovirus infection or of a disorder associated with secondary PRCA, a computed tomography scan of the chest should be performed to rule out a thymoma, which would have potential therapeutic implications [18]. Our patient had anemia with low reticulocyte count, normal platelets counts, and the leukopenia was considered immunological in origin. We confirmed PRCA in our patient by the bone marrows biopsy, where there was a marked reduction in erythroid lineage with normal megakaryopoiesis and increased granulopoiesis. A viral screen for a possible etiology, autoimmune screen, and CECT to exclude thymoma was done to rule out other secondary causes for PRCA other than RA in this patient.

Pathogenesis of PRCA is heterogeneous where it involves immune dysfunction with antibodies directed against erythroid precursor cells or erythropoietin, or due to T cell-mediated suppression of erythropoiesis [4, 18]. The immunoglobulin (IgG)-mediated inhibition of hemoglobin synthesis or complement-binding and direct cytotoxic effects on erythroblasts were found in patients with PRCA, wherein some patients, the inhibitory antibodies were directed against erythropoietin [20]. In addition, PRCA can be mediated by major histocompatibility complex (MHC) unrestricted effector-target cell recognition, as erythroid progenitors progressively lose expression of MHC class I and, thus, become susceptible to destruction by NK-type cells [18]. The final result of all these mechanisms is anemia, with bone marrow examination revealing a complete absence of erythroblasts but normal granulocytic and megakaryocytic series [21].

Depending on the cause, the course of PRCA can be acute and self-limiting or chronic with rare spontaneous remissions [18]. Otherwise, treating PRCA varies with the etiological factors; however, the treatment of choice is immunosuppression for primary acquired autoimmune PRCA or secondary PRCA refractory to other therapy [10, 16]. The therapeutic plan usually focuses on the sequential use of various immunosuppressive therapies, including corticosteroids, cyclophosphamide, cyclosporin A and antithymocyte globulin, splenectomy, and plasmapheresis until remission is obtained [4]. Recently, the efficacies of the anti-CD20 monoclonal antibody, rituximab, and anti-CD52 monoclonal antibody, alemtuzumab, to induce remissions of therapy-resistant PRCA have also been reported [4]. PRCA secondary to autoimmune/collagen vascular disorders may respond to therapy specific to managing those disorders [10]. The goal of treatment is to induce remission to attain an average hemoglobin concentration with the recovery of erythropoiesis, without any requirement for transfusion and avoiding problems associated with transfusions; a partial response is the attainment of transfusion independence with a low but clinically acceptable hemoglobin concentration [4, 10]. Our patient has been treated with prednisolone 40 mg daily, which tailed off over 1 month, with concurrent treatment of cyclosporin A and hydroxychloroquine, where she attained complete recovery within 2 months.

The clinical outcome of PRCA is variable from complete remission to fatal, and it depends upon the cause and the types of treatment [5, 17, 22–24]. Some patients with PRCA show no response to treatment [5]. At the same time, continuous immunosuppression is associated with an increased risk of infection and malignancy; therefore, adequate prevention of infection will be essential [24]. Nevertheless, our patient did not develop any complications due to immunosuppressive therapy.

The final diagnosis of our patient was long-standing deforming rheumatoid arthritis complicated with PRCA, with immune-mediated leukopenia and vasculitis. The challenges in the diagnosis were to exclude the possible other etiologies of the PRCA, leukopenia, generalized lymphadenopathy, and venous thrombosis in an unusual site. Extensive lymphadenopathy and venous thrombosis were also reported in patients with rheumatoid arthritis [25]. A nationwide cohort study in Taiwan recruiting nearly 30,000 RA patients has shown a 3.36-fold increased risk of deep venous thrombosis and a 2.07-fold increase in pulmonary embolism than in patients without RA [26]. A study done in the UK has also concluded a similar result [27]. Lymphadenopathy is also a clinical manifestation of RA, indicating the disease activity [28]. Some studies found that the overall frequency of lymphadenopathy in patients with RA is as high as 82%, mainly involving axillary lymph nodes [29]. Lymphadenopathy at unusual sites has also been reported in patients with RA, contributing to venous thrombosis at unusual sites due to vascular compression [30]. Venous thrombosis involving the axillary vein in our patients may be attributed to the possible lymphadenopathy. We excluded the possibility of hematological malignancy by doing a lymph node biopsy and repeating the bone marrow biopsy as both showed no evidence of leukemia or lymphoma. The thrombophilia screen also excluded antiphospholipid syndrome, and the overlapping systemic lupus erythematosus (SLE) was also excluded as the 2012 Systemic Lupus International Collaborating Clinics (SLICC) criteria was not satisfied [31]. Hence, these manifestations were thought to be immune-mediated due to rheumatoid arthritis by the multidisciplinary team involving a hematologist.

In our patient, the major drawback to starting immunosuppressive drugs was leukopenia [32]. Nevertheless, she showed a marked improvement after starting cyclosporin A, hydroxychloroquine, and steroids. Cyclosporin A has proven to be the drug of choice for PRCA, and the second-line therapy for PRCA includes antithymocyte globulin (ATG) and cyclophosphamide [3, 33, 34]. She was transfusion dependent for nearly 16 months, but she was completely free of blood transfusion after the treatment. Her joint deformities were fixed but minimally interfering with daily living activities, and her constitutional symptoms were also improved.

## Conclusion

PRCA is a disabling illness that may lead to transfusion-dependent anemia, causing transfusion-related complications. RA is a known cause for secondary acquired PRCA due to the disease itself or the RA treatments. Patients with PRCA present with symptoms of severe anemia, which may be less pronounced because of adaptation with gradual hemoglobin drop. Blood analyses and bone marrow examination can diagnose PRCA, and investigating for a secondary cause is essential because of its therapeutic importance. Sometimes the diagnosis of PRCA secondary to RA can be challenging because of the presence of leukopenia, lymphadenopathy, and venous thrombosis at unusual sites due to immunologic and vasculitic phenomena. The treatment of choice for PRCA is immunosuppression, which includes multiple modalities, and the goal of treatment is to recover erythropoiesis without any requirement for transfusion and avoid problems associated with transfusions. The clinical outcome of PRCA can be variable through early diagnosis, and commencing treatments is crucial for a better prognosis and to prevent irreversible complications, and most evidence-based treatment will significantly improve the outcome.

## Data Availability

The authors confirm that the data supporting the findings of this study are available within the article.

## Abbreviations

RA: Rheumatoid arthritis
PRCA: Pure red cell aplasia
ACR: American College of Rheumatology
CECT: Contrast-enhanced computed tomography
Hb: Hemoglobin
MCV: Mean corpuscular volume
MCHC: Mean corpuscular hemoglobin concentration
NSAID: Non-steroidal antiinflammatory drugs
HIV: Human immunodeficiency virus
RF: Rheumatoid factor
Anti-CCP: Anti-cyclic citrullinated peptide
ANA: Antinuclear antibody
Anti dsDNA: Anti double-stranded deoxyribonucleic acid
TNF: Tumor necrosis factors
ATG: Antithymocyte globulin
